# Novel SNP Combination for Predictive Osteoporotic Diagnosis

**DOI:** 10.3390/ijms262211117

**Published:** 2025-11-17

**Authors:** Julia V. Sopova, Olga A. Krasnova, Polina I. Semenova, Julia D. Kryukova, Giomar V. Vasileva, Anna S. Zhuk, Olga M. Lesnyak, Vitaliy V. Karelkin, Irina E. Neganova

**Affiliations:** 1 Laboratory of Molecular Medicine, Institute of Cytology, Russian Academy of Sciences, St. Petersburg 194064, Russia; 2 Center for Transgenesis and Genome Editing, Saint-Petersburg State University, St. Petersburg 199034, Russia; 3 Center of Genetic Reprogramming and Gene Therapy, Institute of Cytology, Russian Academy of Sciences, St. Petersburg 194064, Russia; 4 Institute of Applied Computer Science, ITMO University, St. Petersburg 197101, Russia; 5 Department of Family Medicine, North-Western State Medical University Named After I.I. Mechnikov, St. Petersburg 195298, Russia; 6 Traumatology and Orthopedics Department, Vreden National Medical Research Center of Traumatology and Orthopedics, St. Petersburg 195427, Russia

**Keywords:** osteoporosis, GPCR, *ADRB2*, *FSHR*, *TSHR*, polymorphism, SNP combination, osteodifferentiation

## Abstract

Osteoporosis is a multifactorial disease, the pathogenesis of which is caused by a complex interaction of genetic, hormonal, and metabolic factors. The challenges of early diagnosis highlight the need to identify genetic predictors to prevent bone mineral density (BMD) loss. Given the critical role of G-protein-coupled receptors (GPCRs) in bone development and remodeling, we investigated osteoporosis-associated single-nucleotide polymorphisms (SNPs) within GPCR genes using next-generation sequencing of patient cohorts. Subsequent screening via Sanger sequencing identified three SNPs for further analysis: rs1991517 in the thyroid-stimulating hormone receptor gene (*TSHR*), rs6166 in the follicle-stimulating hormone receptor gene (*FSHR*), and rs1042713 in the β2-adrenergic receptor gene (*ADRB2*). Our results reveal a significant association between osteoporosis and a specific homozygous genotype combination (TSHR rs1991517 CC, FSHR rs6166 AA, and ADRB2 rs1042713 AA). The functional impairment in osteodifferentiation was further validated in patient-derived cell lines harboring this triple-SNP combination. Thus, this study is the first to identify a specific combination of GPCR gene polymorphisms that may serve as a predictive biomarker for osteoporosis in early genetic screening.

## 1. Introduction

Osteoporosis is a widespread systemic disease that affects approximately 200 million people worldwide. The characteristic features of this disease include decreased bone mineral density (BMD), alterations in bone architecture, and, as a result, an increased risk of fractures [1]. The number of diagnosed cases is growing globally, thus raising the treatment costs and placing a significant burden on healthcare systems [2]. Women over 50 years old are at the highest risk of developing osteoporosis, yet reliable early diagnosis remains unavailable [3].

In the Russian Federation, the incidence of osteoporosis-related fractures is similar to other regions of the world [4]. According to the statistics, the total number of proximal femur fractures in Russia in 2015 exceeded 126,000, and by 2050, this number is projected to increase by 70%, partly due to population aging [5]. By 2035, the total number of osteoporotic fractures is expected to reach 730,000 cases per year [4].

Currently, the diagnosis of osteoporosis is based on patient medical history, physical examination, and BMD measurement. The latter is considered to be the most reliable prognostic method for assessing the risk of osteoporotic fractures [6,7]. Nowadays, extensive genetic research is being conducted to understand the genetic causes of decreased BMD and osteoporosis development [8,9], since bone mineral density is a heritable trait [10]. Next-generation sequencing (NGS) enables the targeted screening of genetic variants that might be associated with osteoporosis [11,12]. In most cases, osteoporosis arises not from a single gene mutation, but as a possible result of the cumulative effect of many common single-nucleotide polymorphisms (SNPs) [13]. In the elderly population, where osteoporosis is prevalent, the impact of other comorbidities like hypertension [14] or diabetes [15] becomes more prominent. The association between these comorbid conditions may be mediated, at least in part, by the impaired functioning of G-protein-coupled receptors (GPCRs)—proteins involved in all fundamental biological processes. Epidemiological studies have shown that individuals with hypertension have a higher prevalence of osteoporosis and an increased risk of fractures [16]. Treatment with Angiotensin-converting enzyme inhibitors (ACEIs) or Angiotensin receptor blockers is associated with a higher BMD and a reduced fracture risk in hypertensive patients. Furthermore, the use of beta-blockers, which antagonize β2-AR, has been associated in some epidemiological studies with a reduction in fracture risk [17,18]. Patients with T2DM have an increased risk of osteoporosis and fractures, despite often having normal or high BMDs [19].

GPCRs play a critical role in bone development and remodeling [20,21,22]. Genome-wide association studies (GWASs) on different ethnic groups have shown that SNPs in at least 26 GPCR genes are associated with a decreased BMD and osteoporosis progression [23,24]. The study of osteoporosis-associated SNPs in GPCR genes holds not only significant prognostic importance but has profound therapeutic significance, since GPCRs serve as the target for over 30% of all currently marketed pharmaceuticals [25]. For instance, drugs like teriparatide and strontium ranelate exert their anti-osteoporotic effects through interactions with the PTH type 1 receptor (PTHR1) and calcium-sensing receptor (CaSR), respectively [26,27].

Despite the large amount of data generated by NGS analysis, information regarding the role of specific SNPs or their combinations in the development of osteoporosis in individual patients remains insufficient. Osteoporosis is a classic polygenic disease, where the pathological phenotype results from the interactions between multiple genes. Currently, various genetic tests are available to assess predisposition to osteoporosis, detecting mutations in a range of genes, including

- *COL1A1* (encoding the α1-chain of type I collagen),
- *CYP19A1* (encoding aromatase, responsible for converting testosterone to estradiol),
- *ESR1* (encoding the estrogen receptor), and others listed in Supplementary Table S1 [28,29,30,31,32,33,34,35,36,37].

However, these mutations are often associated with hereditary forms of other severe diseases (e.g., the rs1555571755 variant in the *COL1A1* gene is linked to osteogenesis imperfecta [28], while the rs2414096 variant in *CYP19A1* is associated with polycystic ovary syndrome [31]) and might be prognostic for severe forms of early-onset osteoporosis. To find new prospective SNP combinations for osteoporosis prediction, we turned to the GPCR genes that have great impact on osteogenesis.

The aim of this study was to identify the combination of known osteoporosis-associated SNPs in GPCR genes in osteoporotic patients and analyze the impact of their combinations on the osteodifferentiation of patient-specific cell lines. For the first time, the significant role of a combination of homozygous alleles of the *TSHR* (rs1991517) CC, *FSHR* (rs6166) AA, and *ADRB2* (rs1042713) AA alleles on the competence of osteodifferentiation in patient-specific cell lines was demonstrated.

## 2. Results

### 2.1. Identification of Osteoporosis-Associated SNPs in GPCR Genes

To search for SNPs in GPCR genes associated with osteoporosis, we have isolated genomic DNA from six osteoporotic patients’ venous blood samples and performed whole-genome sequencing. Additionally, we obtained the whole-genome sequence of the genomic DNA isolated from the venous blood samples of two patients with occasional fractures and no osteoporosis diagnosis. The resulting sequencing data were analyzed to identify known pathogenic variants associated with osteoporosis development. Gene variants were selected based on studies by Luo et al. [23] and from our previous work, Domnina et al. [24]. The list of selected SNPs is presented in Supplementary Table S2 [38,39,40,41,42,43,44,45,46,47,48,49,50,51,52,53,54,55,56,57,58,59,60,61,62,63,64,65,66,67,68,69,70].

This NGS analysis revealed that the samples from the six studied osteoporotic patients differ by the combinations of specific osteoporosis-related SNPs in GPCR genes. The majority of the detected SNPs were located in non-coding regions and therefore most likely influence gene expression efficiency. Only one-third of the detected SNPs were missense mutations (Table 1). These missense variants caught our specific attention, since amino acid substitution may alter the functional activity of the given protein by affecting folding, ligand binding, localization, and post-translational modification, etc. These effects are most prominent in the homozygous state, so we focused on homozygous missense mutations, found in osteoporotic patients. Particularly, three missense SNP variants attracted our attention, being the most abundant in three genes, namely, the *FSHR* (rs6166), *TSHR* (rs1991517), and *ADRB2* (rs1042713) genes. These SNPs were found in the triple-homozygous combination in one out of six patients, and we have earlier shown the impact of the homozygous *ADRB2* rs1042713 AA allele on the osteoblastogenesis of patient-specific cell lines [22].

The *FSHR* gene encodes for the receptor of a follicle-stimulating hormone that plays an important role not only in reproductive processes, but also in bone metabolism. The *FSHR* c.2039G > A variant leads to an S680N amino acid substitution and was present in three out of six patients (Table 1) in the homozygous state (AA). According to the literature, this SNP influences FSHR protein responsiveness to exogenous FSH, and AA homozygosity is associated with a reduced bone mineral density (BMD) [55].

The receptor of the thyroid-stimulating hormone (TSHR) is found on the surface of thyroid follicular cells, but is also expressed in osteoblasts and osteoclasts [71,72]. The *TSHR* c.2181G > C variant causes an E727D substitution and was found in all six patients, with four being homozygous (CC) (Table 1). Previous studies have shown that this SNP is associated with a susceptibility to autoimmune thyroid diseases, and CC homozygotes typically exhibit decreased femoral neck BMD [61].

The β2-adrenergic receptor, coded by the *ADRB2* gene, mediates physiological responses to catecholamines and is found on the surface of various cell types, including osteoblasts [73,74]. The *ADRB2* c.46G > A variant results in a G16R substitution and was present in four of six patients, two of whom were homozygous (AA) (Table 1). Previously, it was shown that the homozygous AA genotype had significantly higher frequency in Spanish women with reduced BMDs [75], which is in line with our recent results [22]. Controversially, Korean studies report only slightly increased BMDs in AA homozygotes [41], reflecting the impact of ethnic group on phenotype manifestation.

Thus, the co-occurrence of these SNPs in three GPCR genes which potentially contribute to BMD reduction may have a combined effect on the development of osteoporosis. To establish reliable associations of this gene combination with osteoporosis predisposition, we have increased the sample size and genotyped 120 osteoporotic patients using allele-specific real-time PCR and Sanger sequencing.

### 2.2. The Combination of Triple-Homozygous FSHR (rs6166 AA), TSHR (rs1991517 CC), and ADRB2 (rs1042713 AA) Alleles Is Frequent in Patients with Osteoporosis

**Figure 1:**
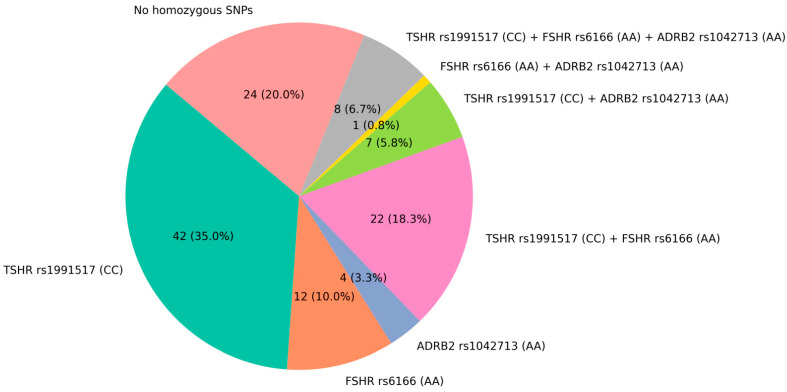
Distribution of homozygous genotypes for the selected SNPs in the *FSHR* (rs6166), *TSHR* (rs1991517), and *ADRB2* (rs1042713) genomes of osteoporotic patients.

To expand the data on the selected SNP combination, we have analyzed genomic DNA from 120 patients diagnosed with osteoporosis for the presence of selected SNPs in the *FSHR* (rs6166), *TSHR* (rs1991517), and *ADRB2* (rs1042713) genes using allele-specific real-time PCR. Ten randomly selected patients were genotyped by Sanger sequencing, showing a 100% concordance with results obtained by allele-specific real-time PCR. All patients were divided into groups bearing one homozygous SNP, two homozygous SNPs, or a combination of all three homozygous SNP alleles (presented in Figure 1). As expected, we did observe the high abundance of *TSHR* rs1991517 CC homozygotes (35%) among osteoporotic patients, but, surprisingly, we have found the high percentage of triple homozygotes (*TSHR* rs1991517 (CC), *FSHR* rs6166 (AA), and *ADRB2* rs1042713 (AA), 6.7%)) and the presence of only one double *FSHR* rs6166 (AA) and *ADRB2* rs1042713 (AA) homozygote (0.8%) (Figure 1).

According to the dbSNP database (https://www.ncbi.nlm.nih.gov/snp, accessed on 26 August 2025), in the European population the estimated frequencies of the *TSHR* rs1991517 C allele is 0.91, *FSHR* rs6166 A allele = 0.55, and *ADRB2* rs1042713 A allele = 0.37. In our group of osteoporotic patients the estimated frequency of the *TSHR* rs1991517 C allele is 0.80, *FSHR* rs6166 A allele = 0.60, and *ADRB2* rs1042713 A allele = 0.37. These values do not significantly differ from the European population data, so the genotype frequencies observed in this patient cohort might be representative of the global population.

**Figure 2:**
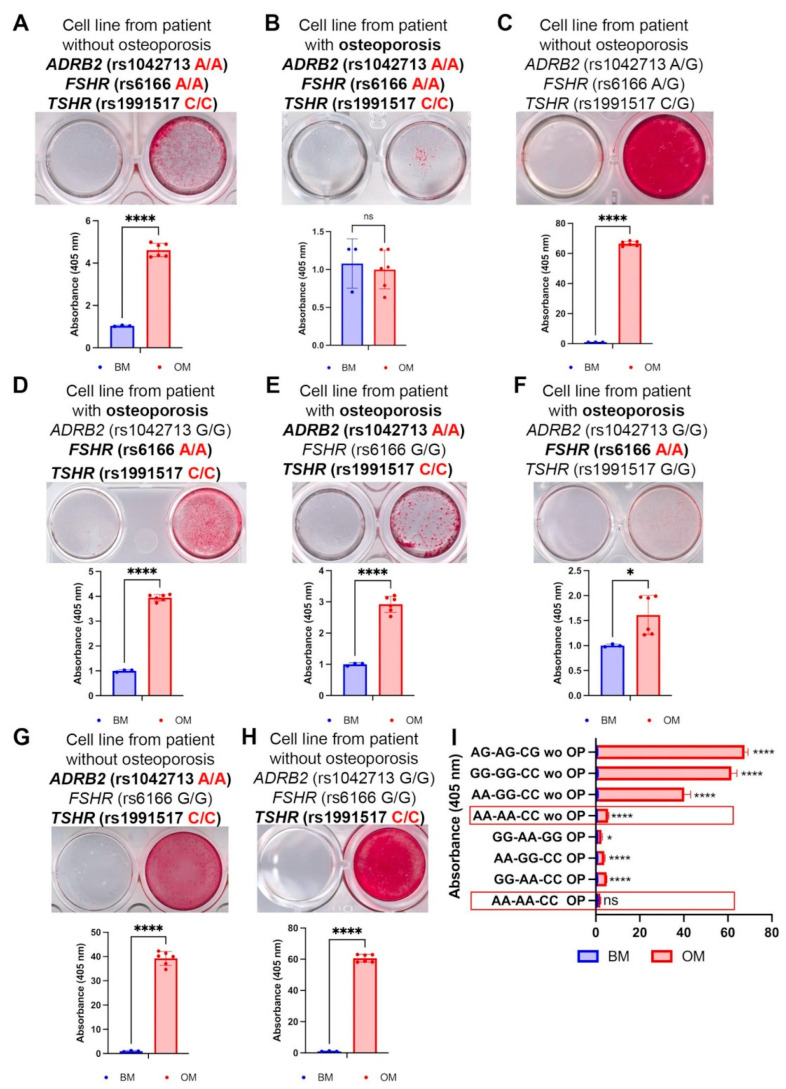
(**A**–**H**) Osteogenic differentiation of patient-specific cell lines, bearing different combinations of SNPs in the *ADRB2* (rs1042713), *FSHR* (rs6166), and *TSHR* (rs1991517) genes. Cells were stained with Alizarin Red on the 21st day of differentiation. (**I**) Summarized quantified Alizarin Red staining in patient-specific cell lines. The first NN stands for *ADRB2* (rs1042713), the second for *FSHR* (rs6166), and the third for *TSHR* (rs1991517). The cell lines from patients with triple-homozygous SNPs are framed. Data are shown as mean ± SD, *n* > 3, with significant differences indicated with asterisks (*—*p* < 0.05, ****—*p* < 0.0001, and ns—not significant). Abbreviations: BM—basic medium and OM—osteogenic medium.

To study the impact of each SNP on osteoblastogenesis, we have obtained the patient-specific mesenchymal stem cell (MSC) lines from the bone samples of osteoporotic patients and the control patients with occasional fractures. The obtained cell lines were maintained in the osteogenic media for 21 days, and then the matrix mineralization was evaluated using Alizarin Red staining. The typical results of this staining in various genotype combinations are presented in Figure 2.

Triple-homozygous cells (*ADRB2* rs1042713 [AA], *FSHR* rs6166 [AA], and *TSHR* rs1991517 [CC]) from a patient with an occasional fracture exhibited slight but significant Alizarin Red staining after osteoinduction (Figure 2A), indicating a severely impaired mineralization capacity. This impairment was even more pronounced in a cell line with the same genotype from an osteoporotic patient, which showed a complete absence of staining (Figure 2B), suggesting a total block of osteogenesis in vitro. In contrast, control cells from a non-osteoporotic, triple-heterozygous patient (*ADRB2* rs1042713 [A/G], *FSHR* rs6166 [A/G], and *TSHR* rs1991517 [C/G]) demonstrated intense Alizarin Red staining (Figure 2C).

The presence of two homozygous SNPs (*FSHR* rs6166 [AA] and *TSHR* rs1991517 [CC]) in cells from osteoporotic patients decreased staining (Figure 2D). Similarly, the combination of *TSHR* rs1991517 (CC) and *ADRB2* rs1042713 (AA) weakened osteodifferentiation (Figure 2E), as did the single-homozygous *FSHR* rs6166 (AA) polymorphism (Figure 2F). This indicates that all three studied polymorphisms contribute to impaired osteogenic differentiation.

In MSCs from a patient with an occasional non-osteoporotic fracture, the combination of *ADRB2* rs1042713 (AA) and *TSHR* rs1991517 (CC) (Figure 2G) slightly reduced osteogenic differentiation compared to MSCs with only *TSHR* rs1991517 (CC) (Figure 2H) or the triple-heterozygous profile (Figure 2C). This suggests a synergistic negative effect of these polymorphisms.

Overall, quantification revealed that MSCs from a non-osteoporotic patient with the triple-homozygous SNP profile exhibited a staining pattern similar to that of osteoporotic patient lines, while one osteoporotic line with this genotype demonstrated a complete inhibition of osteoblastogenesis in vitro (Figure 2I). This implies that these SNPs play an essential role in regulating osteogenic differentiation and may enhance a pre-existing impairment.

**Figure 3:**
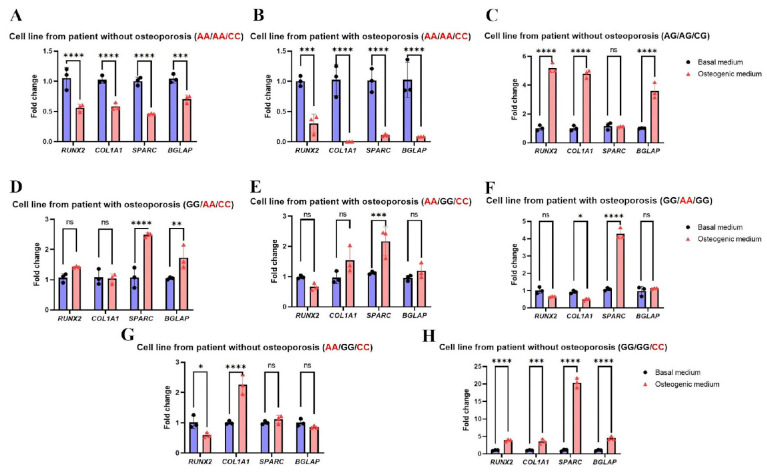
(**A**–**H**) Analysis of the gene expressions of osteogenic differentiation markers (*RUNX2*, *COL1A1*, *SPARC*, and *BGLAP*) by qPCR in patient-specific cell lines with different combinations of SNPs in the *FSHR* (rs6166), *TSHR* (rs1991517), and *ADRB2* (rs1042713) genes. Cells were harvested on day 7 of differentiation. Circle denotes basal medium, triangle—osteogenic medium. Data are presented as mean ± SD (*n* = 3). Significant differences are indicated with asterisks (ns—not significant, *—*p* < 0.05, **—*p* < 0.01, ***—*p* < 0.001, and ****—*p* < 0.0001).

The Alizarin Red staining data, which reflects the effectiveness of osteogenic differentiation, were corroborated by a qPCR analysis of key osteogenic markers (*RUNX2*, *COL1A1*, *SPARC*, and *BGLAP*). These genes are involved in all stages of differentiation, from initiation to matrix maturation and mineralization. A pronounced downregulation of these markers was found in cells with a triple-homozygous SNP profile (*TSHR* rs1991517 CC, *FSHR* rs6166 AA, and *ADRB2* rs1042713 AA) (Figure 3A, B). The extent of downregulation depended on the clinical diagnosis, ranging from a 2-fold decrease in cells from a patient without osteoporosis (Figure 3A) to an almost-complete suppression in cells from an osteoporotic patient (Figure 3B). In contrast, cells from a patient with a triple-heterozygous SNP profile showed the upregulation of genes involved in all osteogenic differentiation stages (Figure 3C). Specifically, we observed a 4.5-fold increase in the expression of *RUNX2*, a master transcription factor for the initiation of osteogenic differentiation [76]; a 4.5-fold upregulation of *COL1A1*, which encodes the pro-α1(I) chain of Type I collagen—the most abundant bone matrix protein required for matrix assembly [77]; and a significant increase in the mRNA *BGLAP* level [78], which encodes osteocalcin, a highly abundant non-collagenous protein responsible for matrix mineralization.

The combination of two homozygous SNPs (*FSHR* rs6166 (AA) and *TSHR* rs1991517 (CC)) in cells from an osteoporotic patient was associated with enhanced matrix maturation and mineralization (Figure 2D). This is supported by the upregulation of *SPARC* (encoding osteonectin, essential for collagen fibril assembly and extracellular matrix formation [79]) and *BGLAP* (reflecting the mineralization stage), consistent with the Alizarin Red staining results (Figure 3D). Conversely, the blocked osteogenic differentiation seen in Figure 2E was confirmed by qPCR. In cells with the *TSHR* rs1991517 (CC) and *ADRB2* rs1042713 (AA) combination, we observed no significant upregulation of *RUNX2*, *COL1A1*, or *BGLAP* (Figure 3E). The upregulation of *SPARC* alone appears insufficient to drive effective osteogenic differentiation (Figure 3E). This finding is supported by the qPCR analysis of MSCs from a patient with only the *FSHR* rs6166 (AA) variant (Figure 3F). In these cells, we observed only *SPARC* upregulation, with no change in *RUNX2* or *BGLAP* expression and a downregulation of *COL1A1*. This gene expression profile is consistent with the impaired mineralization shown by the Alizarin Red staining (Figure 2F).

Surprisingly, in cells from a patient with an occasional (non-osteoporotic) fracture carrying the *TSHR* rs1991517 (CC) and *ADRB2* rs1042713 (AA) combination, only *COL1A1* expression was enhanced (Figure 3G) despite the pronounced mineralization detected by Alizarin Red staining (Figure 2G).

The presence of the *TSHR* rs1991517 (CC) variant alone did not impair osteogenic differentiation at the transcriptional level, as all examined markers were significantly upregulated (Figure 3H). The extent of matrix mineralization, assessed by Alizarin Red staining (Figure 2H), was comparable to that observed in patient-specific cells with the triple-heterozygous SNP profile (Figure 2C). Thus, the qPCR data support the Alizarin Red staining findings and demonstrate that triple-homozygosity for *TSHR* rs1991517 CC, *FSHR* rs6166 AA, and *ADRB2* rs1042713 AA severely impairs osteogenic differentiation, affecting the process from transcription to matrix mineralization.

## 3. Discussion

Osteoporosis is one of the leading causes of disability worldwide. As the pharmaceutical industry ramps up the production of both anabolic and antiresorptive drugs to cure the disease, osteoporosis is no longer a disease of the elderly. Fracture rates in the working-age population are rising globally. At the same time, early diagnosis of osteoporosis remains poor and existing methods primarily diagnose the disease when changes in bone density and architecture have already occurred. All this highlights the need to develop methods for diagnosing osteoporosis in its early stages.

As osteoporosis manifests itself as a polygenic disease, there have been studies that have uncovered SNPs in numerous genes associated with low BMD, and other bone defects are well known. Additionally, the number of GWAS and NGS studies in various ethnic populations is growing, often showing significant differences in the identified genes between European and Asian populations. Importantly, these studies are often isolated and not confirmed by patient-specific cell line studies, complicating the interpretation and translation of results into personalized medicine.

In our study, we conducted whole-genome sequencing of the blood samples of osteoporosis patients. Whole-genome sequencing of a small subset of patients first revealed the SNP combination in three genes, namely, *TSHR* rs1991517 (CC), *FSHR* rs6166 (AA), and *ADRB2* rs1042713 (AA). Next, we expanded the number of the patient cohort to 120 individuals and demonstrated that the occurrence of this triple-SNP combination in this cohort is still significant (6.7%). The employment of patient-specific MSC lines with this SNPs combination revealed their incompetence to fully complete osteogenic differentiation in vitro, supporting our idea that the homogeneous combination of SNPs in these genes, namely, *TSHR* rs1991517 (CC), *FSHR* rs6166 (AA), and *ADRB2* rs1042713 (AA), might indeed serve as a prognostic combination for osteoporosis.

However, other frequent GPCR gene variants may also influence the osteoporosis progression in the patient-specific cell lines that were included in the present study. We found, for example, an SNP in the *P2RY2* gene (rs2511241 C/T). Interestingly, the TT homozygote in this gene has been associated with osteoporosis risk in the Dutch population [59]. The frequency of the rs2511241 T allele reaches 0,91 in the European population; however, in our study we did not find any rs2511241 CC genotype, even in the control patient group, so the prognostic value of this SNP might be relatively low. Another variant is *LEPR* (rs1137100 A/G), with the AA homozygote being associated with osteoporosis progression in Chinese Mulao populations [70] but not in Danish populations [80]. These contradictory data derived from large patient cohorts may indicate the necessity of evaluating the functional role of these SNPs in osteodifferentiation, particularly in patient-derived cell cultures. The homozygous variant *GNRH1* rs6185 (CC), associated with osteoporosis [51], was found in four patients, and might be used as prognostic SNP, but this requires additional study. In addition, two more variants were associated with a lower BMD—an inactivating variant in the CaSR gene at rs1801725 (TT homozygote) and a functional variant in the GIPR gene at rs1800437 (CC homozygote) [39,67]. However, we did not find these homozygous SNPs in our study. The dopamine receptor D2 (*DRD2*) gene variant rs1800497 (TT homozygote) was associated with a lower BMD in patients diagnosed with chronic schizophrenia in Taiwan [45]; however, the relevance of this SNP in other population groups might be limited.

The process of osteodifferentiation can be categorized into at least three core stages, such as initiation, matrix synthesis/maturation, and mineralization. We have earlier shown that osteoporotic patient-derived MSCs with various SNP combinations have defects on different stages of osteoblast maturation and differentiation [21]. Furthermore, we previously showed that the *ADRB2* rs1042713 (AA) variant exhibits low osteogenic potential due to the preservation of the proliferative activity of MSCs obtained from osteoporotic patients bearing this SNP, which further leads to the ablation of the matrix synthesis and mineralization [22]. In this study, we demonstrated for the first time the potential significance of the combination of three polymorphisms in GPCR genes, each of which individually introduces only a moderate contribution to the development of the disease. The SNPs discussed in this paper are non-synonymous, meaning that they lead to changes in the amino acid sequence and the activity of the encoded receptors. These SNPs are found in GPCRs that are widely expressed throughout the human body and are involved in endocrine regulation. The most affected osteoporotic patient group is postmenopausal women. This group often exhibits elevated FSH serum levels [55], a high susceptibility to hyperthyroidism [71], and elevated catecholamine levels associated with widespread hypertension [81]. These endogenous agonists lead to more pronounced aberrant receptor activity in the presence of the aforementioned SNPs. All the discussed GPCRs are known to facilitate osteoclastogenesis via canonical pathways (e.g., RANK), thereby promoting bone resorption [82,83,84]. The data presented herein, however, suggests that the activity of bone-forming osteoblasts is also impaired by these SNPs. Consequently, triple-homozygous SNP combinations, namely, the *TSHR* rs1991517 (CC), *FSHR* rs6166 (AA), and *ADRB2* rs1042713 (AA), appear to affect osteoblast function as well, a phenomenon that should be elucidated further on patient-specific cell lines. This combination had the greatest impact on all stages of the osteodifferentiation of patient-specific cell lines, shown by the Alizarin Red staining and qPCR analyses. The triple-homozygous profile in MSCs from the patient with occasional, non-osteoporotic fractures results in an Alizarin Red staining pattern similar to MSCs from osteoporotic patients. Therefore, while we suggest that each SNP may contribute to the impairment of osteogenic differentiation to a different extent, the combination of the three homozygous variants may become the most effective early diagnosis marker, before the start of disease manifestation.

It has been suggested that developing a robust genetic test employing only one or two SNPs with high allele frequencies results in a low predictive value [85]. On the other hand, analyzing a large number of SNPs simultaneously, as is common in many GWAS studies, makes it difficult to isolate and understand the contribution of each individual variant. Notably, the combination of the two SNPs in the *FSHR* (rs6166) and *TSHR* (rs1991517) genes might also be predictive, since it was found in 25% of cases independently of *ADRB2* rs1042713 presence, though this needs further evaluation. Nevertheless, the impact of the combination of two SNPs (*TSHR* rs1991517 (CC) and *FSHR* rs6166 (AA)) in the homozygous state on osteodifferentiation was not as drastic as it was for the triple combination. Further work in this area requires the expansion of sample size and the inclusion of premenopausal women together with functional analyses of the influence of individual SNPs on the efficiency of osteogenic differentiation of patient-specific mesenchymal stem cells from patients with varying comorbidies, sex, and age. Our next goal is to identify the most optimal combination of polymorphisms for early diagnostic purposes, since the low efficacy of current early-stage osteoporosis diagnostics means that the disease often goes undetected until a critical decrease in BMD has occurred.

One of the study limitations is related to the patient cohort, which consisted of postmenopausal women with osteoporosis, most of whom already had a history of fractures. Therefore, the question of whether or not premenopausal women would pose this triple-SNP combination as a prognostic marker remains open. Further genotyping to identify selected homozygous combinations in premenopausal women, followed by the longitudinal monitoring of their BMD, could help to validate this SNP combination for predictive osteoporosis diagnostics at its very beginning. Next, patient-specific cell lines are genetically heterogeneous. Therefore, to delineate the role of each individual polymorphism (or their combinations), genetic editing may be necessary. The genotyping of cohorts with high risks of osteoporosis and comorbid diseases (hypertension and diabetes), and subsequential data comparisons will allow for the determination of the optimal SNP combination for early diagnosis with the highest accuracy.

## 4. Materials and Methods

### 4.1. Patients

Venous blood samples for the whole-genome sequencing study were collected from six patients diagnosed with osteoporosis and two patients with occasional fractures, which were used as controls. Samples of the femur were harvested during surgery at Vreden National Medical Research Center of Traumatology and Orthopedics, Saint-Petersburg, Russia from 44 patients with osteoporosis diagnosis. Venous blood samples were also obtained from these patients and used for Sanger sequencing and the detection of SNPs using real-time PCR. The femur samples and venous blood samples from 11 patients with occasional fractures and no diagnosed osteoporosis were used for genotyping and the selection of control cell lines. The whole-genome sequencing, Sanger sequencing, detection of SNPs using real-time PCR, and clinical research protocols were approved by the local ethics committee of the A.A. Vreden National Medical Research Center of Traumatology and Orthopedics (St. Petersburg, Russia) and complied with the ethical principles of the Declaration of Helsinki. All participants provided written informed consent.

To expand the patient cohort, additional venous blood samples were obtained from 76 patients with osteoporosis diagnoses at Clinical Rheumatology Hospital No. 25. The Sanger sequencing and detection of SNPs using a real-time PCR study protocol was approved by the local ethics committee of Clinical Rheumatology Hospital No. 25 (St. Petersburg, Russia) in accordance with the Declaration of Helsinki. All participants provided written informed consent. The demographic data of the patient cohorts are presented in Supplementary Table S3.

### 4.2. Whole-Genome Sequencing Protocol

Genomic DNA was isolated from whole blood samples using the Blood and Cell Culture DNA Midi Kit (Qiagen, Hilden, Germany). One microgram of each DNA sample was used for whole-genome library preparation. DNA fragmentation was performed using the M220 Focused-ultrasonicator with microTUBE-50 tubes (Covaris, Woburn, MA, USA), targeting a 350 bp fragment size. Whole-genome DNA libraries were prepared using TruSeq DNA PCR-Free Library Preparation Kits (Illumina, San Diego, CA, USA).

All library preparation steps and subsequent procedures were conducted using supplementary reagent kits according to the manufacturers’ protocols. The resulting libraries were quantified using the KAPA Library Quantification Kit for Illumina sequencing platforms (KAPA Biosystems, Wilmington, MA, USA) and sequenced on the Illumina HiSeq 4000 system (Illumina, San Diego, CA, USA).

### 4.3. Read Alignment and Variant Calling

A quality assessment of the Illumina short reads was performed using FastQC [86], and aggregate quality metrics were visualized with MultiQC [87]. Reads were aligned to the GRCh38 reference genome using the BWA-MEM (v0.7.17) with default parameters [88]. Resulting alignments were converted to BAM, sorted, and indexed using SAMtools. Duplicate reads were identified and flagged with Picard MarkDuplicates (v2.27.3) (http://broadinstitute.github.io/picard, accessed on 12 November 2025). The base quality score recalibration was carried out using GATK (v4.2.6.1) [89] with the BaseRecalibrator and ApplyBQSR, incorporating known sites from the dbSNP155 database (https://www.ncbi.nlm.nih.gov/snp/, accessed on 12 November 2025).

Variant calling was performed using the GATK HaplotypeCaller in GVCF mode, followed by the merging of the GVCFs with CombineGVCFs and joint genotyping using GenotypeGVCFs. The variant dataset was filtered using the VariantRecalibrator and ApplyVQSR, with known SNPs from dbSNP155. Only biallelic SNPs were retained for downstream analysis. Variant annotation was performed using the Ensembl Variant Effect Predictor (VEP) v111 [90].

### 4.4. Genomic DNA Isolation, Sanger Sequencing, and Detection of SNPs Using Real-Time PCR

Genomic DNA for the sequencing of selected alleles of the FSHR, TSHR, and ADRB2 genes was isolated using the “DNA-sorb-V” kit (AmpliSens, Moscow, Russia) from the patients’ blood samples according to the manufacturer’s protocol.

The amplification of nucleotide sequences and Sanger sequencing were performed using the following oligonucleotide primers:

FSHR for 5′-TTTGTGGTCATCTGTGGCTGC-3′

FSHR rev 5′-CAAAGGCAAGACTGAATTATCATT-3′

TSHR for 5′-CCATTCCTCTATGCTATTTTCAC-3′

TSHR rev 5′-CCGTTTGCATATACTCTTCTG-3′

ADRB2 for 5′-CACCTGCCAGACTGCGCGCC-3′

ADRB2 rev 5′-GAAGTCCAAAACTCGCACCAG-3′

The detection of SNPs in the *TSHR* (rs1991517), *FSHR* (rs6166), and *ADRB2* (rs1042713) genes was performed with RT-PCR kits (DNK-Sintez, Moscow, Russia) according to the manufacturer’s protocol.

### 4.5. Cell Isolation and Cultivation

Cell isolation was performed as described in Krasnova et al. [22]. Briefly, bone samples were cut into small fragments and washed with phosphate-buffered saline (PBS) (Merck, Rahway, NJ, USA) supplemented with 100 U/mL penicillin–streptomycin (Thermo Fisher Scientific, Waltham, MA, USA). Next, the bone fragments were incubated in collagenase II (Gibco, Waltham, MA, USA) solution in DMEM (1.5 mg/mL) for 30 min at 37 °C and 5% CO_2_. After incubation, the collagenase II solution was replaced with collagenase IV (Gibco, Waltham, MA, USA) solution in DMEM (1.5 mg/mL) for overnight incubation at 37 °C and 5% CO_2_. The next day, the bone fragments were washed with PBS and placed in tissue culture flasks (TPP, Trasadingen, Switzerland), the surfaces of which were scraped in advance in a low-glucose DMEM (1 g/mL D-glucose) (Thermo Fisher Scientific, Waltham, MA, USA) with 10% fetal bovine serum (FBS) (Thermo Fisher Scientific, Waltham, MA, USA) and 100 U/mL penicillin–streptomycin (Thermo Fisher Scientific, Waltham, MA, USA) at 37 °C and 5% CO_2_.

### 4.6. Osteogenic Differentiation and Calcium Deposit Staining with Alizarin Red

The patient-derived cells were grown on 24-well plates (TPP, Trasadingen, Switzerland) up to 80% of confluence. Then, growth media were replaced with osteogenic media (DMEM with 1 g/mL D-glucose, 10% FBS, 100 U/mL penicillin–streptomycin, 10 mM β-glycerophosphate, 200 μM L–Ascorbic acid, and 100 nM dexamethasone (all Sigma Aldrich, St. Louis, MO, USA)). Cells were refed every 3–4 days. After 21 days of osteogenic differentiation induction, MSCs were stained with Alizarin Red S (Merck Millipore, Rahway, NJ, USA). Photometric quantification of Alizarin Red S concentrations was performed according to the protocol listed in [91]. The mean and standard deviation of at least three independent experiments are presented. A Student’s t-test was used for testing the differences between groups. *p*-values < 0.05 were considered statistically significant.

### 4.7. Quantitative Polymerase Chain Reaction (qPCR) Analysis of Osteogenic Differentiation Markers’ Gene Expressions

Cells were grown on 12-well plates (TPP, Trasadingen, Switzerland) in basal medium (control) and osteogenic medium for 7 days and then harvested for RNA isolation. The Biolabmix kit RUplus (Biolabmix, Novosibirsk, Russia) was used for RNA isolation according to the manufacturer’s instructions. cDNA was synthesized using the Revertaid H Minus Strand cDNA Synthesis Kit (Thermo Fisher Scientific, Waltham, MA, USA), following the manufacturer’s instructions. Real-time PCR experiments were performed using the BioMaster HS-qPCR SYBR Blue (2×) (Biolabmix, Russia) in the Bio-Rad CFX Opus-96 real-time system (Bio-Rad Laboratories, Hercules, CA, USA), according to the kit’s attached protocol using primers listed in Table 2. The gene expression in cells cultured in the basal medium was applied as a control and the expression of target genes was normalized to the *GAPDH* gene and calculated using the ^ΔΔ^Ct method. All amplifications were run at three technical replicates, and all experiments were performed at three biological repeats. Statistical analysis was carried out using the one-way ANOVA, and GraphPad Prism 9 software (GraphPad Software Inc., Version 9.5.1 (528), La Jolla, CA, USA) was used for data analysis.

## Figures and Tables

**Table 1:** Osteoporosis-associated SNPs found in patients’ genomes by NGS sequencing.

Gene	rsID	Type	Patient #1	Patient #2	Patient #3	Patient #4	Patient #5	Patient #6
***LEPR***	**rs1137100**	**Missense variant**	**A/G**	**A/G**	**A/G**	**GG**	**AA**	**A/G**
***FSHR***	**rs6166**		**AA**	**AA**	**GG**	**GG**	**GG**	**AA**
***CASR***	**rs1801725**		**G/T**	**GG**	**GG**	**G/T**	**G/T**	**G/T**
***ADRB2***	**rs1042713**		**G/A**	**GG**	**GG**	**G/A**	**AA**	**AA**
***CALCR***	**rs1801197**		**TT**	**CC**	**TT**	**TT**	**TT**	**T/C**
***GNRH1***	**rs6185**		**CC**	**CC**	**GG**	**CC**	**C/G**	**CC**
***P2RY2***	**rs2511241**		**TT**	**C/T**	**TT**	**TT**	**TT**	**TT**
***DRD2***	**rs1800497**		**CC**	**C/T**	**CC**	**C/T**	**C/T**	**TT**
***TSHR***	**rs1991517**		**CC**	**G/C**	**CC**	**G/C**	**CC**	**CC**
***GIPR***	**rs1800437**		**G/C**	**GG**	**GG**	**GG**	**GG**	**G/C**
*NPY2R*	rs2880415	Synonymous variant	TT	TT	C/T	C/T	C/T	TT
*NPY2R*	rs6857715	Intron variant	TT	C/T	C/T	C/T	TT	TT
*OPRM1*	rs4870268		TT	T/C	T/C	CC	CC	T/C
*OPRM1*	rs9479769		TT	T/C	T/C	CC	CC	T/C
*OPRM1*	rs1998221		TT	T/C	T/C	CC	CC	T/C
*CALCR*	rs2051748		A/G	AA	AA	A/G	AA	A/G
*CALCR*	rs2051748		A/G	AA	AA	A/G	AA	A/G
*LGR4*	rs7936621		G/A	G/A	GG	G/A	GG	G/A
*MTNR1B*	rs3781638		TT	TT	G/T	TT	G/T	TT
*ADGRD1*	rs1880842		GG	GG	GG	GG	GG	GG
*LGR4*	rs10835153	Intergenic variant	TT	TT	A/T	TT	TT	TT
*MC4R*	rs17782313		TT	TT	T/C	TT	TT	TT
*FZD1*	rs2232157	5′prime UTR variant	GG	T/G	T/G	T/G	T/G	TT
*FZD1*	rs2232158		GG	T/G	T/G	T/G	T/G	TT
*CALCR*	rs1042138	3′prime UTR variant	GG	G/A	GG	GG	GG	GG

Note: missense variants are shown in **bold** and homozygous missense variants associated with osteoporosis are highlighted in grey.

**Table 2:** Primers used for qPCR analysis.

Gene	Primers
*RUNX2*	Forward GAG TGG ACG AGG CAA GAG T Reverse GGG TTC CCG AGG TCC ATC TA
*COL1A1*	Forward GAC CTA AAG GTG CTG CTG GAG Reverse CTT GTT CAC CTC TCT CGC CA
*SPARC*	Forward GGC CTG GAT CTT CTT TCT C Reverse CCC ACA GAT ACC TCA GTC A
*BGLAP*	Forward GGC AGC GAG GTA GTG AAG AG Reverse CTG GAG AGG AGC AGA ACT GG
*GAPDH*	Forward TGC ACC ACC AAC TGC TTA GC Reverse GGC ATG GAC TGT GGT CAT GAG

## Data Availability

The original contributions presented in this study are included in the article/Supplementary Materials. Further inquiries can be directed to the corresponding author(s).

## Supplementary Materials

The following supporting information can be downloaded at https://www.mdpi.com/article/10.3390/ijms262211117/s1.
