# Supplementary material for: Novel SNP Combination for Predictive Osteoporotic Diagnosis
Source: Int J Mol Sci. 2025 Nov 17;26(22):11117. doi: 10.3390/ijms262211117 (PMC12652954; doi:10.3390/ijms262211117)
Supplement: Supplementary file 1 [file ijms-26-11117-s001.zip › ijms-3948359-supplementary.pdf]

Supplementary Table 1. Specific SNPs used in commercially available diagnostic tests for osteoporosis.

Gene	SNP	Protein function	Association with other diseases	Reference
COL1A1	rs1555571755<br>rs1800012	α1-chain of type I collagen; component of bone extracellular matrix	Osteogenesis imperfecta	[28, 29]
CYP19A1	rs2414096<br>rs936306	Aromatase, catalyzes conversion of testosterone to estradiol	Polycystic ovary syndrome	[30, 31]
ESR1	rs2234693<br>rs9340799	Estrogen receptor	Preeclampsia	[32]
IL6	rs1800795	Interleukin-6, mediates inflammatory response	Cardiovascular pathologies	[33]
LRP5	rs4988321<br>rs3736228	Low-density lipoprotein (LDL) transmembrane receptor	Rheumatoid arthritis	[34]
RANKL	rs9594738<br>rs9594759	RANK ligand (receptor activator of nuclear factor kappa-B ligand); stimulates osteoclast activity, osteoprotegerin antagonist	Delayed permanent tooth eruption	[35]
TNFRSF11B	rs3134069<br>rs4355801<br>rs3102735	Osteoprotegerin; regulates bone remodeling by inhibiting osteoclasts, thereby increasing bone strength	Osteoarthritis	[36]
VDR	rs1544410<br>rs10735810	Vitamin D receptor	Pregnancy complications linked to vitamin D deficiency	[37]

28. Lu Y., Ren X., Wang Y., et al. Mutational and structural characteristics of four novel heterozygous C-propeptide mutations in the proα1(I) collagen gene in Chinese osteogenesis imperfecta patients. Clin Endocrinol (Oxf). 2014; 80(4):524-31. doi: 10.1111/cen.12354.

29. Braga V., Mottes M., Mirandola S., et al. Association of CTR and COLIA1 alleles with BMD values in peri- and postmenopausal women. Calcif Tissue Int. 2000; 67:361–366. doi: 10.1007/s002230001160.

30. Hong X., Hsu Y.H., Terwedow H., et al. CYP19A1 polymorphisms are associated with bone mineral density in Chinese men. Hum Genet. 2007; 121(3-4):491-500. doi: 10.1007/s00439-006-0303-1.

31. Sharma, P.; Kaur, M.; Khetarpal, P. CYP19 gene rs2414096 variant and differential genetic risk of polycystic ovary syndrome: A systematic review and meta-analysis. Gynecol. Endocrinol. 2021, 37, 126–131. https://doi.org/10.1080/09513590.2020.1813274.

32. Mondockova V., Adamkovicova M., Lukacova M., et al. The estrogen receptor 1 gene affects bone mineral density and osteoporosis treatment efficiency in Slovak postmenopausal women. BMC Med Genet 2018; 19:174 https://doi.org/10.1186/s12881-018-0684-8

33. Ji Y.F., Jiang X., Li W., Ge X. Impact of interleukin-6 gene polymorphisms and its interaction with obesity on osteoporosis risk in Chinese postmenopausal women. Environ Health Prev Med 2019; 24:48 doi: 10.1186/s12199-019-0803-y

34. Kruk M., Ralston S.H., Albagha O.M. LRP5 Polymorphisms and response to risedronate treatment in osteoporotic men. Calcif Tissue Int. 2009; 84(3):171-9. doi: 10.1007/s00223-008-9207-5.

35. Zhu D.L., Chen X.F., Hu W.X., et al. Multiple Functional Variants at 13q14 Risk Locus for Osteoporosis Regulate RANKL Expression Through Long-Range Super-Enhancer. J Bone Miner Res. 2018; 33(7):1335-1346. doi: 10.1002/jbmr.3419.

36. Roshandel D., Holliday K.L., Pye S.R., et al; EMAS Study Group. Genetic variation in the RANKL/RANK/OPG signaling pathway is associated with bone turnover and bone mineral density in men. J Bone Miner Res. 2010; 25(8):1830-8. doi: 10.1002/jbmr.78.

37. Yureneva S.V., Donnikov A.E., Bordakova E.V., Yakushevskaya O.V., Smetnik A.A., Trofimov D.Yu. Clinical and prognostic significance of molecular genetic factors in postmenopausal osteoporosis. Osteoporosis and Bone Diseases. 2015;18(1):3-6. (In Russ.) https://doi.org/10.14341/osteo201513-6

Supplementary Table 2. Genetic variants of GPCR family proteins associated with osteoporosis development.

Gene	SNP	Minor Allele Frequency (MAF)	Protein function	References
Metabotropic Glutamate Receptors				
CASR	rs1801725	0.26	Calcium-sensing receptor that responds to extracellular calcium concentration changes and plays a key role in calcium homeostasis.	[38,39]
Rhodopsin-like Receptors				
GABBR1	rs3025642	0.17	Component of the heterodimeric GABA receptor formed by GABBR1 and GABBR2.	[40]
ADRB2	rs1042713	0.49	Beta-adrenergic receptors that mediate catecholamine-induced adenylate cyclase activation.	[41]
CNR2	rs2501431<br>rs3003336<br>rs2229579<br>rs4237	0.50<br>0.50<br>0.28<br>0.49	Heterotrimeric receptor for endocannabinoid 2-arachidonoylglycerol that mediates adenylate cyclase inhibition.	[42-44]
DRD2	rs1800497	0.46	Dopamine receptor whose activity inhibits adenylate cyclase.	[45]
DRD4	rs1800955	0.50	Dopamine receptor responsible for neuronal signaling in the brain's mesolimbic system - the region regulating emotions and complex behaviors.	[46]
MC4R	rs17782313<br>rs17700633<br>rs121913566<br>rs747681609<br>rs187152753<br>rs121913562<br>rs13447329	0.37<br>0.46<br><0.01<br><0.01<br>0.01<br><0.01<br><0.01	Receptor of the leptin-melanocortin regulatory pathway.	[47, 48]
Melatonin Receptors				
MTNR1B	rs3781638	0.50	High-affinity melatonin receptor	[49]
Growth Hormone Secretagogue Receptors				
GHSR	rs495225	0.48	Ghrelin receptor that stimulates growth hormone secretion	[50]
Gonadotropin-Releasing Hormone Receptors				
GNRHR	rs6185	0.49	GnRH receptor that stimulates secretion of LH and FSH	[51]
Neuropeptide Y Receptors				
NPY2R	rs2880415<br>rs6857715	0.50	Receptor for neuropeptide Y and peptide YY	[52]
Chemokine Receptors				
CCR2	rs1799864	0.33	MCP-1 receptor mediating calcium mobilization and adenylate cyclase inhibition	[53]
Opioid Receptors				
OPRM1	rs9479769<br>rs4870268<br>rs1998221	0.49<br>0.49<br>0.49	Endogenous opioid receptor for β-endorphin and endomorphin	[54]
Follicle-Stimulating Hormone Receptors				
FSHR	rs6166	0.50	FSH receptor activating PI3K-AKT and ERK1/2 pathways	[55]

Leucine-Rich Repeat-Containing GPCRs				
LGR4	rs7936621<br>rs10835153<br>rs587777005	0.50<br>0.49<br><0.01	R-spondin receptor initiating Wnt/β-catenin signaling	[56-58]
Purine Receptors				
P2RY2	rs2511241	0.16	ATP/UTP receptor activating phosphatidylinositol-calcium system	[59]
Relaxin Family Peptide Receptors				
RXFP2	rs121918303	0.02	Relaxin receptor stimulating adenylate cyclase via G-proteins	[60]
Thyroid-Stimulating Hormone Receptors				
TSHR	rs1991517	0.22	TSH receptor activating adenylate cyclase	[61]
Adhesion GPCRs				
ADGRD1	rs1880842	0.50	Orphan receptor	[62]
Frizzled/Taste2 Receptors				
FZD1	rs2232157<br>rs2232158	0.49<br>0.49	Wnt protein receptor in canonical Wnt/β-catenin pathway	[63]
Secretin Family Receptors				
CALCR	rs1801197<br>rs2051748<br>rs1042138	0.50<br>0.50<br>0.46	Calcitonin receptor activating adenylate cyclase	[64, 65]
CRHR1	rs9303521	0.50	CRH/UCN receptor inhibiting CACNA1H calcium channel	[66]
GIPR	rs1800437	0.29	GIP receptor activating adenylate cyclase	[67]
PTHR1	rs1138518	0.48	PTH receptor activating adenylate cyclase and PI-calcium system	[68]
Other 7-Transmembrane Domain Receptors				
WLS	rs2772300	0.39	Regulates Wnt protein sorting and secretion through feedback mechanisms	[69]
LEPR	rs1137100<br>rs2767485	0.40<br>0.36	Leptin (LEP) hormone receptor that mediates central and peripheral effects through JAK2/STAT3 and MAPK/FOS signaling pathways upon ligand binding	[70]

Note: Minor Allele Frequency values were taken from Ensembl database (https://www.ensembl.org/index.html, accessed on 28.08.2025).

38. Lorentzon M, Lorentzon R, Lerner UH, Nordstrom P. Calcium sensing receptor gene polymorphism, circulating calcium concentrations and bone mineral density in healthy adolescent girls. Eur. J. Endocrinol. 2001;144:257–261. doi: 10.1530/eje.0.1440257.
39. Di Nisio A., Rocca M.S., Ghezzi M., et al. Calcium-sensing receptor polymorphisms increase the risk of osteoporosis in ageing males. Endocrine. 2018; 61:349–352. doi: 10.1007/s12020-017-1429-8
40. Liang X, Wu C, Zhao H, et al. Assessing the genetic correlations between early growth parameters and bone mineral density: A polygenic risk score analysis. Bone. 2018; 116:301-306. doi: 10.1016/j.bone.2018.08.021
41. Lee H.J., Kim H., Ku S.Y., et al. Association between polymorphisms in leptin, leptin receptor, and β-adrenergic receptor genes and bone mineral density in postmenopausal Korean women. Menopause. 2014;21(1):67-73. doi: 10.1097/GME.0b013e31829366ed.
42. Woo J.H., Kim H., Kim J.H., Kim J.G. Cannabinoid receptor gene polymorphisms and bone mineral density in Korean postmenopausal women. Menopause. 2015; 22:512–519. doi: 10.1097/GME.0000000000000339.
43. Yamada Y., Ando F., Shimokata H. Association of candidate gene polymorphisms with bone mineral density in community-dwelling Japanese women and men. Int. J. Mol. Med. 2007; 19:791–801.
44. Karsak M., Cohen-Solal M., Freudenberg J., et al. Cannabinoid receptor type 2 gene is associated with human osteoporosis. Hum. Mol. Genet. 2005;14:3389–3396. doi: 10.1093/hmg/ddi370.
45. Chiang T. I., Lane, H. Y., Lin, C. H. D2 dopamine receptor gene (DRD2) Taq1A (rs1800497) affects bone density. Scientific reports 2020; 10(1):13236. doi: 10.1038/s41598-020-70262-0
46. Yamada Y., Ando F., Niino N., Shimokata H. Association of a polymorphism of the dopamine receptor D4 gene with bone mineral density in Japanese men. J. Hum. Genet. 2003;48:629–633. doi: 10.1007/s10038-003-0090-7.

47. Garg G., Kumar J., McGuigan F.E., et al. Variation in the MC4R gene is associated with bone phenotypes in elderly Swedish women. PLoS One. 2014;9(2):e88565. doi: 10.1371/journal.pone.0088565.
48. Farooqi I.S., Yeo G.S., Keogh J.M., et al. Dominant and recessive inheritance of morbid obesity associated with melanocortin 4 receptor deficiency. J Clin Invest. 2000;106(2):271-9. doi: 10.1172/JCI9397
49. Li Y., Zhou J., Wu Y., et al. Association of osteoporosis with genetic variants of circadian genes in Chinese geriatrics. Osteoporos Int. 2016;27(4):1485-1492. doi: 10.1007/s00198-015-3391-8.
50. Dennison E.M., Syddall H.E., Jameson K.A., et al; Hertfordshire Cohort Study Group. A study of relationships between single nucleotide polymorphisms from the growth hormone-insulin-like growth factor axis and bone mass: the Hertfordshire cohort study. J Rheumatol. 2009;36(7):1520-6. doi: 10.3899/jrheum.081061
51. Iwasaki H., Emi M., Ezura Y., et al. Association of a Trp16Ser variation in the gonadotropin releasing hormone signal peptide with bone mineral density, revealed by SNP-dependent PCR typing. Bone. 2003;32(2):185-90. doi: 10.1016/s8756-3282(02)00949-3
52. Chun E.H., Kim H., Suh C.S., et al. Polymorphisms in neuropeptide genes and bone mineral density in Korean postmenopausal women. Menopause. 2015; 22(11):1256-63. doi: 10.1097/GME.0000000000000454
53. Eraltan H., Cacina C., Kahraman O. T., et al. MCP-1 and CCR2 Gene Variants and the Risk for Osteoporosis and Osteopenia. Genetic Testing and Molecular Biomarkers 2012;16(4):229–233. doi:10.1089/gtmb.2011.0216
54. Lu S., Zhao L.J., Chen X.D., et al. Bivariate genome-wide association analyses identified genetic pleiotropic effects for bone mineral density and alcohol drinking in Caucasians. J Bone Miner Metab. 2017; 35(6):649-658. doi: 10.1007/s00774-016-0802-7.
55. Rendina D., Gianfrancesco F., De Filippo G., et al. FSHR gene polymorphisms influence bone mineral density and bone turnover in postmenopausal women. Eur J Endocrinol. 2010; 163(1):165-72. doi: 10.1530/EJE-10-0043.
56. Shi S.Q., Li S.S., Zhang X.Y., et al. LGR4 Gene Polymorphisms Are Associated With Bone and Obesity Phenotypes in Chinese Female Nuclear Families. Front Endocrinol (Lausanne). 2021; 12:656077. doi: 10.3389/fendo.2021.656077
57. Zhang X., Deng H.W., Shen H., Ehrlich M. Prioritization of osteoporosis-associated genome-wide association study (GWAS) single-nucleotide polymorphisms (SNPs) using epigenomics and transcriptomics. JBMR Plus. 2021;5(5):e10481. doi: 10.1002/jbm4.10481
58. Styrkarsdottir U., Thorleifsson G., Sulem P. et al. Nonsense mutation in the LGR4 gene is associated with several human diseases and other traits. Nature 2013;497:517–520 doi: 10.1038/nature12124
59. Wesselius A., Bours M. J., Henriksen Z., et al. Association of P2Y(2) receptor SNPs with bone mineral density and osteoporosis risk in a cohort of Dutch fracture patients. Purinergic signalling 2013;9(1):41–49. doi: 10.1007/s11302-012-9326-3
60. Ferlin A., Pepe A., Gianesello L., et al. Mutations in the insulin-like factor 3 receptor are associated with osteoporosis. Journal of bone and mineral research : the official journal of the American Society for Bone and Mineral Research 2008;23(5):683–693. doi: 10.1359/jbmr.080204
61. van der Deure W.M., Uitterlinden A.G., Hofman A., et al. Effects of serum TSH and FT4 levels and the TSHR-Asp727Glu polymorphism on bone: the Rotterdam Study. Clin Endocrinol (Oxf). 2008;68(2):175-81. doi: 10.1111/j.1365-2265.2007.03016.x.
62. Morris, J.A., Kemp, J.P., Youlten, S.E. et al. Author Correction: An atlas of genetic influences on osteoporosis in humans and mice. Nat Genet 51, 920 (2019). https://doi.org/10.1038/s41588-019-0415-x
63. Zhang Y., Kuipers A. L., Yerges-Armstrong L. M., et al. Functional and association analysis of frizzled 1 (FZD1) promoter haplotypes with femoral neck geometry. Bone 2010;46(4):1131–1137. doi: 10.1016/j.bone.2009.12.026
64. Masi L., Becherini L., Colli E., et al. Polymorphisms of the calcitonin receptor gene are associated with bone mineral density in postmenopausal Italian women. Biochem Biophys Res Commun. 1998;248(1):190-5. doi: 10.1006/bbrc.1998.8880.
65. Lee HJ., Kim SY., Kim G.S. et al. Fracture, bone mineral density, and the effects of calcitonin receptor gene in postmenopausal Koreans. Osteoporos Int 2010;21:1351–1360. doi: 10.1007/s00198-009-1106-8
66. Rivadeneira F., Styrkársdottir U., Estrada K., et al; Genetic Factors for Osteoporosis (GEFOS) Consortium. Twenty bone-mineral-density loci identified by large-scale meta-analysis of genome-wide association studies. Nat Genet. 2009;41(11):1199-206. doi: 10.1038/ng.446.
67. Torekov S.S., Harsløf T., Rejnmark L., et al. A functional amino acid substitution in the glucose-dependent insulinotropic polypeptide receptor (GIPR) gene is associated with lower bone mineral density and increased fracture risk. J Clin Endocrinol Metab. 2014;99(4):E729-33. doi: 10.1210/jc.2013-3766.
68. Abdi S., Almiman A. A., Ansari M. G. A., et al. PTHR1 Genetic Polymorphisms Are Associated with Osteoporosis among Postmenopausal Arab Women. BioMed research international 2021; 2993761. doi: 10.1155/2021/2993761
69. Roshandel D., Thomson W., Pye S. R., et al; EMAS Study Group Polymorphisms in genes involved in the NF-κB signalling pathway are associated with bone mineral density, geometry and turnover in men. PloS one 2011;6(11):e28031. doi: 10.1371/journal.pone.0028031
70. Ye G., Huang Y., Yin L., et al. Association between LEPR polymorphism and susceptibility of osteoporosis in Chinese Mulao people. Artif Cells Nanomed Biotechnol. 2022;50(1):10-16. doi: 10.1080/21691401.2021.2020279.

Supplementary Table 3. The demographic data of patients' cohorts.

	Osteoporotic patients		Patients with occasionally fractures	
Race	Caucasian			
Sex	114 women (postmenopausal)	6 men	6 women (postmenopausal)	5 men
Age (median)	70	59.5	58.5	66
Fracture presence	97 with at least one fracture<br>17 without fractures	All 6 with at least one fracture	All 6 with at least one fracture	All 5 with at least one fracture
